# HIV treatment in Guinea-Bissau: room for improvement and time for new treatment options

**DOI:** 10.1186/s12981-020-0259-6

**Published:** 2020-02-04

**Authors:** S. Jespersen, F. Månsson, J. Lindman, C. Wejse, C. Medina, Z. J. da Silva, DdS Te, P. Medstrand, J. Esbjörnsson, B. L. Hønge

**Affiliations:** 1 Department of Infectious Diseases, Aarhus University Hospital, Palle Juul-Jensens Boulevard 99, 8200 Aarhus N, Denmark; 2 Bandim Health Project, Indepth Network, Apartado 861, 1004 Bissau Codex, Guinea-Bissau; 3 Department of Translational Medicine, Lund University, J Waldenströms gata 35, 205 02 Malmö, Sweden; 4 Department of Clinical Sciences Lund, Division of Infection Medicine, Lund University, BMC F12, 221 84 Lund, Sweden; 5 GloHAU, Center for Global Health, Department of Public Health, Aarhus University, Bartholins Allé 2, 8000 Aarhus, Denmark; 6 National HIV Programme, Ministry of Health, Bissau, Apartado 861, 1004 Bissau Codex, Guinea-Bissau; 7 National Public Health Laboratory, CP 1013 Bissau, Guinea-Bissau; 8 Nuffield Department Medicine, University of Oxford, Old Road Campus, Headington, Oxford OX3 7BN UK

**Keywords:** HIV-1, HIV-2, Dual infection, Antiretroviral treatment, Guinea-Bissau, West Africa

## Abstract

Despite advances in the treatment quality of HIV throughout the world, several countries are still facing numerous obstacles in delivering HIV treatment at a sufficiently high quality, putting patients’ lives in jeopardy. The aim of this status article is to give an overview of HIV treatment outcomes in the West African country, Guinea-Bissau, and to assess how newer treatment strategies such as long-acting injectable drugs or an HIV cure may limit or stop the HIV epidemic in this politically unstable and low-resource setting. Several HIV cohorts in Guinea-Bissau have been established and are used as platforms for epidemiological, virological, immunological and clinical studies often with a special focus on HIV-2, which is prevalent in the country. The Bandim Health Project, a demographic surveillance site, has performed epidemiological HIV surveys since 1987 among an urban population in the capital Bissau. The Police cohort, an occupational cohort of police officers, has enabled analyses of persons seroconverting with estimated times of seroconversion among HIV-1 and HIV-2-infected individuals, allowing incidence measurements while the Bissau HIV Cohort and a newer Nationwide HIV Cohort have provided clinical data on large numbers of HIV-infected patients. The HIV cohorts in Guinea-Bissau are unique platforms for research and represent real life in many African countries. Poor adherence, lack of HIV viral load measurements, inadequate laboratory facilities, high rates of loss to follow-up, mortality, treatment failure and resistance development, are just some of the challenges faced putting the goal of “90–90–90″ for Guinea-Bissau well out of reach by 2020. Maintaining undetectable viral loads on treatment as a prerequisite of a cure strategy seems not possible at the moment. Thinking beyond one-pill-once-a-day, long-acting antiretroviral treatment options such as injectable drugs or implants may be a better treatment option in settings like Guinea-Bissau and may even pave the way for an HIV cure. If the delivery of antiretroviral treatment in sub-Saharan Africa in a sustainable way for the future should be improved by focusing on existing treatment options or through focusing on new treatment options remains to be determined.

## Introduction

Despite advances in the treatment quality of HIV throughout the world, several countries are still facing numerous obstacles in delivering HIV treatment at a sufficiently high quality, putting patients’ lives in jeopardy [1, 2]. In many West African countries, the level of pre-treatment drug resistance exceeds 10%, indicating that programs need to take actions to prevent further development of drug resistance, which may include the transition from non-nucleoside reverse transcriptase inhibitors (NNRTIs) to more robust drug classes [3, 4]. This is especially true for Guinea-Bissau. Poor adherence, high rates of loss to follow-up, lack of HIV viral load (VL) measurements, lack of reliable diagnostic tests for opportunistic infections, inadequate laboratory facilities (including transportation of reagents and service of equipment) as well as low numbers of sufficiently trained staff are just some of the challenges faced [5–10]. The aim of this status article is to give an overview of HIV treatment outcomes in Guinea-Bissau. We also aim to assess how newer treatment strategies such as long-acting injectable drugs or an HIV cure may limit or stop the HIV epidemic in this politically unstable and low-resource setting.

### HIV epidemiology in Guinea-Bissau

**Fig. 1:**
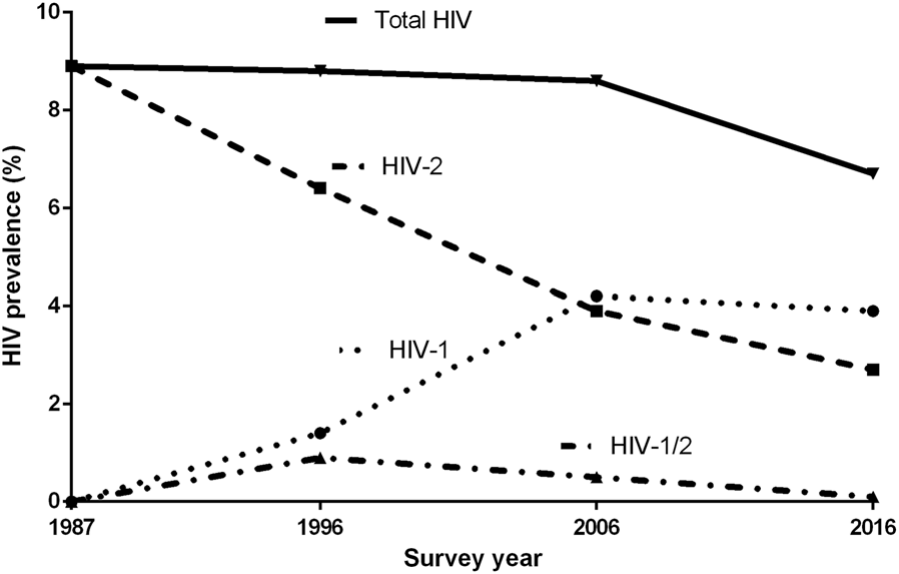
HIV prevalence in four cross-sectional surveys from Bissau, Guinea-Bissau (Adapted from reference [18])

According to the joint United Nations programme on HIV/AIDS (UNAIDS), the HIV prevalence among adults (15–49 years) in Guinea-Bissau was 3.4% (95% CI 2.6–3.8) in 2017 but disparities exist within the country [11]. HIV-2 was discovered two years after HIV-1 [12] and has mainly been restricted to West Africa, where an estimated 1–2 million people are infected with the virus [13]. To evaluate the trends in HIV prevalence and incidence in Guinea-Bissau, the Bandim Health Project (BHP), a demographic surveillance site, has performed epidemiological HIV surveys since 1987 among an urban population in the capital Bissau. In the first BHP survey from 1987, HIV-1 was nonexistent [14]. Two years later the first HIV-1/HIV-2 dual infection was identified in a community cohort study [15]. The prevalence of HIV-1 increased from zero to 4.6% between 1987 and 2006, where it now seems to have stabilized (also the 2016 survey indicated a prevalence of 4.0%, Fig. 1, adapted from reference 18) [16–18]. In contrast to HIV-1, HIV-2 has been steadily decreasing in prevalence from 8.9% in 1987 [14] to 2.8% in 2016 [16–18]. The underlying reasons for this decline in HIV-2 prevalence is not known. However, it is possible that the high HIV-2 prevalence seen during the 1980s was a result of high levels of commercial sex work and blood transfusions during the war of independence from 1963 to 1974 and that the decline in prevalence reflects the low rates of sexual and vertical transmission that is associated with HIV-2 infection [19–21].

### The police cohort

In 1990 an occupational cohort of police officers was initiated in Bissau, supported by the bilateral aid and research program of Swedish SIDA/Sarec. The cohort has been open and prospective, with continuing new recruitment until the civil war broke out in June 1998. Regular follow-up visits were resumed in the year 2000 and new recruitment to the cohort was re-initiated in the year 2003. Until 2011, regular follow-ups were performed, followed by targeted follow-ups since then. Visits were made to police stations in the capital as well as in the interior regions of the country. Follow-up visits were scheduled every 12–18 months, with continuous collection of demographic information and clinical examinations of symptoms related to HIV and sexually transmitted infections. Serological sampling of HIV, HTLV and syphilis were performed. For HIV-infected individuals CD4+ T cell counts have been performed since 1993. By 2011, 4820 police officers had been enrolled, of whom 4817 had a recorded HIV test result. Antiretroviral drugs were provided under the umbrella of the national antiretroviral treatment (ART) programme, initiated in 2005, commencing in the Police cohort in January 2006. The continuous follow-up has enabled analyses of persons seroconverting with estimated times of seroconversion among HIV-1 and HIV-2-infected individuals, allowing incidence measurements [22], as well as unique observations on the natural course of HIV-1 and HIV-2 before treatment became available [23], as well as observations on the interaction between HIV-1 and HIV-2 [24–26]. Collected blood samples has enabled in-depth studies of virological and immunological correlates of HIV-1 and HIV-2-related disease [3, 27–38].

### The Bissau HIV cohort

In 2007, an HIV cohort was set up in Bissau by the BHP and Aarhus University Hospital in Denmark in collaboration with nurses and physicians from Hospital National Simão Mendes (HNSM). HNSM is the main hospital in Guinea-Bissau and is located in Bissau [2]. All HIV-infected patients presenting at the HIV clinic are invited to be enrolled in the cohort. Demographic and clinical data are collected at baseline and at all follow-up visits, together with CD4 + T cell count and routine biochemistry analyses. Due to frequent power cuts in Bissau, plasma and cells are stored in a biobank in Denmark. The cohort currently has recruited > 6500 patients (64% HIV-1, 11% HIV-2, 8% HIV-1/2 and 17% with unknown HIV-type) and is unique because it comprises the world’s largest single-center cohort of HIV-2 and HIV-1/2 dually infected patients. The median age at time of inclusion is 36 years (interquartile range 29–45) with the majority of patients being female (63%). Other infections are co-prevalent in Guinea-Bissau, including tuberculosis [39], HTLV-1 [40–42] and hepatitis [43, 44], creating a unique opportunity to study different co-infections’ effect on disease progression, immune response and response to treatment. The cohort is used as a platform for epidemiological, virological, immunological and clinical studies. The international research collaborations between high- and low-resource settings have helped to identify problems related to delivery of ART [1].

### A nationwide HIV cohort in Guinea-Bissau

As the HIV clinic at HNSM is a referral clinic, and the largest HIV clinic in Guinea-Bissau in terms of number of patients on follow-up, data from this clinic may not always reflect estimates from other HIV clinics in the country. Clinics vary in terms of size, human resources, geography of the area, management, organization and structure. Thus, to address challenges such as mortality and loss to follow-up on a nationwide scale in Guinea-Bissau, a nationwide HIV Cohort was established in 2017 and has now included more than 30,000 patients from nine HIV clinics in the country covering around 90% of all HIV-infected patients enrolled in care in the country. Demographic and clinical data are collected at each patient visit using the same database as for the Bissau HIV Cohort. Comparing patients´ outcome between different clinics, may shed light on the best way of organizing clinics, and ultimately improve the quality of care for HIV-infected individuals in Guinea-Bissau. Furthermore, having a nationwide database can make it possible to see if patients who are thought to be lost to follow-up, in reality have transferred to a different clinic.

### HIV treatment and problems related to ART delivery

In 2005, the Ministry of Health in Guinea-Bissau implemented a national HIV program. During 2007, the program experienced an increase in the number of patients initiating ART, which is free of charge. Unfortunately, the delivery of ART is still facing a multitude of challenges (Table 1). For years, Guinea-Bissau has struggled with political instability, leaving the health sector in a poor state. Frequent HIV clinic relocations and inadequate drug supply leading to treatment interruptions has underscored the need to improve stock management and increase investment in health-care infrastructure and capacity as well as giving the disease a higher priority among policymakers [1]. The HIV-2 prevalence has been declining in Bissau long before ART was introduced and from population-based surveys it seems that ART has had little effect on the pace of the declining HIV-2 prevalence [18, 22, 45–47].

**Table 1:** Challenges related to HIV treatment in Guinea-Bissau

High rates of loss to follow-up
Unawareness of treatment failure
High rates of treatment failure
High rates of resistance
Lack of laboratory facilities and tests
Transport of reagents and keeping the cold chain
Poor adherence
Inadequate ART supply
Patients presenting late
High mortality rates
Lack of HIV related knowledge
Shortage of staff

### Loss to follow-up and adherence

HIV-infected patients in Guinea-Bissau are facing major adherence difficulties and high rates of loss-to-follow-up. A retrospective study among patients in the Bissau HIV Cohort found that 7 years after ART initiation, 56% were lost to the programme, of which 75.9% were lost to follow-up (LTFU), 17.8% died and 6.3% transferred [8]. Main reasons reported for loss to follow-up in Guinea-Bissau are moving (29.1%), travelling (17.5%) and transferring to other clinics (11.7%), suggesting that the majority of the patients LTFU have extended periods of time without, or is no longer receiving ART [48]. A cross-sectional study found that of patients on ART only 14% reported not having missed any dose of ART within the past four days, and conversely only 4% had an adherence of 90% during the last month [49]. This indicate that 86–96% were susceptible to viral failure and disease progression. Moreover, people receiving ART, but who did not attend the clinic as scheduled had a higher risk of non-adherence. Furthermore, non-adherent patients had a lower level of HIV related knowledge suggesting that patients with adherence problems lack the required knowledge to follow the ART regimen, and are thereby in risk of developing viral failure [49, 50].

### Monitoring of treatment and failure

Clinical assessment and laboratory tests play a key role in monitoring the response to treatment and the possible toxicity of ART. According to the WHO Guidelines, VL is recommended as the preferred monitoring approach to diagnose treatment failure. However, CD4 + T cell count and clinical monitoring should be used to diagnose treatment failure if VL is not widely available as is the case in Guinea-Bissau [51]. Previous studies from Guinea-Bissau have also shown that both CD4+ T cell counts (particularly CD4 percentages) and VL are strongly correlated with disease progression in both HIV-1 and HIV-2 infection [10, 24, 33, 52]. Immunological treatment failure is common among patients in the Bissau HIV Cohort. In a study from 2015, immunological treatment failure was detected in 25%, while treatment failure could not be assessed in 37% due to missing CD4+ T cell counts [5], underscoring the problems with lack of laboratory facilities and tests. Recently, a commercial real-time PCR-based HIV-2 VL platform was released (Generic HIV-2, Biocentric, France). However, with the large number of challenges that Guinea-Bissau and many other west African countries face with such assays (e.g. transportation of reagents, keeping the cold chain, stable long-term storage of reagents), developments of other platforms are needed (e.g. an HIV-2 GenXpert platform [Cepheid, USA]) including implementation of point-of-care VL testing [53]. In addition, identification of new biomarkers could provide future opportunities for monitoring of HIV disease progression and ART outcome in HIV infection. The scale-up of effective VL testing is an urgent public health priority and Guinea-Bissau is still lacking behind many other African countries [54].

### Resistance

Poor adherence in a setting where NNRTIs are among the most commonly used ARTs will eventually lead to development of resistance. High rates of NNRTI resistance among HIV-1 infected patients have been seen in studies from the Bissau HIV Cohort and may compromise the future use of this drug class. Genotypes from time of virological failure among patients in the PIONA trial revealed that 73% of patients with virological failure receiving NNRTIs had any nucleoside reverse transcriptase inhibitor (NRTI) or NNRTI mutation [7]. In an observational study of 36 patients with virological failure, resistance testing was performed in 15 patients whereof nine patients (9/15; 60%) had resistance mutations. The most common mutation was K103N, which confers high-level resistance to NNRTIs [6]. Moreover, a recent study with 48 participants demonstrated a 10.4% prevalence of pre-treatment drug resistance to NNRTIs in HIV-1-infected pregnant women in the capital Bissau [4].

HIV-2 is naturally resistant to the NNRTIs [55] and enfuvirtide [56] while integrase inhibitors including dolutegravir are effective against HIV-2 isolates [57–60]. Reduced susceptibility to some protease inhibitors (PIs) has been observed, ritonavir-boosted lopinavir and darunavir being the most active drugs [61, 62]. The World Health Organization recommends dolutegravir containing regimens as the preferred first-line regimen for people living with HIV regardless of HIV type [63]. Resistance-associated mutations against NRTIs, PIs and integrase inhibitors may be selected in HIV-2-infected patients while on therapy [64] but data on HIV-2 resistance in West African patients are scarce [65–68]. However, transmitted drug resistance seems to be rare among HIV-2 infected patients [69]. Algorithms that are used to predict drug resistance in HIV-1 may not be applicable to HIV-2, because the pathways and mutational patterns that lead to resistance differ between the HIV types [70]. In cases of virological failure, HIV-2 resistance is common and the limited HIV-2 therapeutic arsenal and cross-resistance reduces treatment options [67].

### Mortality

In recent time, prognosis of HIV-infected patients has improved to the point where it is possible for a patient to live close to a normal life, if on treatment [71–73]. Several studies on life expectancy in HIV-infected individuals have been performed in Africa, showing life expectancy among patients on ART to be close to those of the background population [74–77]. However, many of these studies are from well-established and well-functioning clinics, and the efficiency of HIV treatment may be lower in other parts of Africa. The success of ART depends on the disease progression at HIV diagnosis, and low CD4+ T cell count at HIV diagnosis has been associated with higher mortality [78]. Almost half of the patients in the Bissau HIV Cohort are presenting with CD4+ T cell counts below 200 cells/µL and additional one quarter with a CD4+ T cell counts below 350 cells/µL [79]. The overall mortality rate was 7.7 per 100 person-years with a higher mortality among patients presenting late and a higher mortality among men [79, 80]. Even though HIV-2 is considered more benign and has fewer pathogenic consequences than HIV-1 for most infected individuals, studies from the Police cohort in Bissau have shown that both HIV-1-infected and HIV-2-infected individuals have a high probability of developing and dying from AIDS without ART [23]. This could partly be explained by the fact that people living with HIV-2 initiate ART later than patients with HIV-1, resulting in higher disease progression and mortality rate [81].

### 90–90–90 goals

To effectively fight the HIV epidemic, UNAIDS have set forth a series of treatment goals. By 2020, 90% of all HIV-infected individuals should be diagnosed. Of these, 90% should be enrolled in ART and 90% of these should be virally suppressed [82]. In low income countries, it is often impossible to correctly asses each branch of the treatment cascade, due to a lack of data, but based on the results from the newest BHP HIV survey [18], and routine analysis from the nationwide HIV Cohort in Guinea-Bissau, preliminary results show that only around 14% of infected individuals are aware of their disease. Of these, only 20% are on treatment, and among patients on treatment in the PIONA trial only 33% were virologically suppressed after one year of ART [7]. These rates are lower than those reported in a review of 89 studies from sub-Saharan Africa in which 78% viral suppression was achieved after 6 months of ART [83]. Maintaining undetectable VL on treatment is at the moment not possible in Guinea-Bissau. Overall, this puts the goal of “90–90–90” for Guinea-Bissau well out of reach by 2020.

### Perspectives regarding long-acting ARTs and HIV cure

In many parts of the World, the extensive use of ART for patients infected with HIV has decreased mortality, improved lives and decreased transmission. The U=U campaign [84] underscoring that patients with undetectable VL in the blood cannot transmit the virus as well as the few side effects from never ART regimens have decreased the need for an HIV cure in countries with well-functioning HIV care and treatment programs. However, in Guinea-Bissau, traditional ART delivery is still not successful due to various obstacles. Maintaining undetectable VL on treatment as a prerequisite of a cure strategy seems not possible at the moment. Thinking beyond one-pill-once-a-day, long-acting ARTs such as injectable drugs or implants may be a better treatment option in settings like Guinea-Bissau and may even pave the way for an HIV cure. Since HIV-2 seems to be susceptible to integrase inhibitors, long-acting Cabotegravir may even be a valid option in all patients irrespective of HIV type, making the problem with unreliable HIV discriminatory rapid tests less important [9, 85, 86]. These methods will also require patients to show up for injections at regular intervals but long-acting ARTs provide invisibility to oneself, one’s partner and household members, and has been preferred in some African studies [87]. Furthermore, long-acting depot progesterone as contraception has been widely used in Africa, making regular injections an acceptable treatment tool.

## Conclusions

Guinea-Bissau still faces numerous challenges in delivering ART at a sufficiently high quality level, and as a result patients’ lives are in jeopardy. The three main HIV cohorts in Guinea-Bissau are unique platforms for research and represent real life in many African countries. Despite difficult working conditions, we have maintained inclusion and follow-up in these cohorts for many years and kept a large number of patients on treatment. The constraints we face are probably also experienced by the many ART facilities in Africa that do not report their data and thereby increasing the risk of publication bias. This may impair further sustainability of ART programs if decisions are based on data not being representative of the general situation. If the delivery of ART in sub-Saharan Africa in a sustainable way for the future should be improved by focusing on existing treatment options or through focusing on new treatment options remains to be determined.

## Data Availability

Not applicable.

## Abbreviations

ART: antiretroviral treatment
BHP: Bandim Health Project
HNSM: Hospital National Simão Mendes
LTFU: lost to follow-up
NRTI: nucleoside reverse transcriptase inhibitor
NNRTIs: non-nucleoside reverse transcriptase inhibitors
PIs: protease inhibitors
VL: viral load
